# Development of Genome-Derived InDel Markers and Genetic Diversity Analysis of *Caragana acanthophylla* in Xinjiang, China

**DOI:** 10.3390/plants15172733

**Published:** 2026-09-07

**Authors:** Yinjuan Yao, Zhenfan Yu, Haojie Bi, Mingzhi Du, Abasi Abudouwaili, Shiji Gong, Junhua Huang

**Affiliations:** 1 College of Forestry and Landscape Architecture, Xinjiang Agricultural University, Urumqi 830052, China; 2 College of Horticulture, Xinjiang Agricultural University, Urumqi 830052, China; 3 College of Landscape Architecture and Tourism Management, Xinjiang Applied Vocational Technical College, Kuitun 833200, China

**Keywords:** *Caragana acanthophylla*, InDel markers, genetic diversity, population structure, isolation by distance, Xinjiang

## Abstract

*Caragana acanthophylla* Kom. is an ecologically important drought-tolerant shrub in Xinjiang, China, but species-specific molecular markers for germplasm characterization remain limited. We sampled 93 individuals from 11 localities representing the currently known distribution of *C. acanthophylla* in Xinjiang. Three individuals per locality (33 in total) were whole-genome resequenced, yielding 2,873,410 high-quality SNPs and 5,679,915 InDels. Genome-wide SNP-based PCA and genetic relationship analysis provided an independent high-resolution assessment of the 33 resequenced individuals. From 34 candidate primer pairs, eight polymorphic InDel markers with stable amplification and clear genotyping profiles were retained and applied to all 93 individuals. The SNP dataset revealed clear regional differentiation and finer locality-associated relationships. Analysis of the same 33 individuals with the eight InDel loci recovered part of this broad pattern, particularly the differentiation of the western YL materials, but showed lower fine-scale resolution. Across all 93 individuals, the InDel panel revealed moderate to low marker-level genetic diversity and detectable regional differentiation. AMOVA attributed 67.00% of the variation to differences among the 11 original sampling localities, while the five exploratory analytical groups showed a similar among-group component (68.37%). The Mantel correlation detected across all 93 individuals (r = 0.801, *p* < 0.001) disappeared after YL was excluded (r = −0.032, *p* = 0.724), indicating that the overall spatial signal was largely driven by the geographic separation of YL. These results support the eight-marker panel as a practical, low-cost tool for preliminary germplasm characterization and broader sample screening, while genome-wide SNP data provide substantially greater resolution for population-level inference.

## 1. Introduction

The genus *Caragana* Fabr. belongs to the family Fabaceae and is mainly distributed from Eastern Europe to temperate Asia. It represents an important shrub group in arid and semi-arid regions. Molecular phylogenetic and chloroplast genomic studies have provided important evidence for species delimitation, phylogenetic relationships and infrageneric classification within *Caragana* [1,2,3,4]. *Caragana acanthophylla* has long adapted to high temperature, drought, saline–alkaline and nutrient-poor habitats, and therefore shows strong stress resistance and environmental adaptability [5]. This species is not only an important shrub component for soil and water conservation, windbreak and sand fixation, and soil improvement in desert and semi-desert ecosystems, but also has potential value for regional ecological restoration, vegetation reconstruction, forage production and landscape greening. It is therefore an important germplasm resource in arid regions [5,6].

Ecologically, *C. acanthophylla* commonly occurs in mountain steppe and desert steppe habitats in Xinjiang. Zhou et al. reported that *C. acanthophylla* and *Stipa sareptana* constitute an important shrub-encroached grassland community type in northern China, with sampling sites mainly involving the western Junggar Basin, the Yili Valley and the northern slope of the Tianshan Mountains in Xinjiang [7]. Species of *Caragana* are frequently used for vegetation restoration and sand stabilization in arid regions, and their seed germination, seed dormancy and seedling growth have received increasing attention [8,9,10]. *Flora of China* records that *C. acanthophylla* occurs in Xinjiang, China, and extends to Kazakhstan, Kyrgyzstan, Tajikistan and Uzbekistan in Central Asia [11]. Based on preliminary field surveys, the 11 field sampling localities included in this study represent the currently known distribution range of *C. acanthophylla* in China. These localities show a discontinuous distribution pattern: Tekes County (TKS) and Gongliu County (GL) are located in the Yili Valley and adjacent mountains of western Xinjiang, whereas the other localities are mainly distributed in the middle-eastern section of the northern Tianshan slope and the southern margin of the Junggar Basin.

Genetic diversity is the foundation for species adaptation to environmental change and for maintaining long-term evolutionary potential. The genetic diversity and genetic structure of plant populations are generally influenced by life-history traits, reproductive systems, distribution range, seed and pollen dispersal ability, and geographic isolation [12,13]. Previous reproductive biological studies have shown that *C. acanthophylla* is highly self-incompatible and has an obligate outcrossing mating system, with bees serving as its primary pollinators [14]. Studies of other Caragana species have shown that geographic isolation, environmental heterogeneity, and historical processes can contribute to population genetic differentiation in arid landscapes [15,16,17,18,19,20]. However, comparable population-level genetic information remains limited for *C. acanthophylla*.

Molecular markers are essential tools for studying plant genetic diversity and population structure. InDel markers are genome-derived insertion/deletion polymorphisms that can be genotyped using relatively simple PCR-based methods and have been widely used in genetic diversity and population structure studies [21,22,23]. With the increasing availability of genomic resources in Caragana, particularly the reference genome of *C. arborescens*, genome-derived InDel marker development has become feasible for *C. acanthophylla* [24]. Recent pan-genome analysis has further revealed extensive genomic and structural variation across Caragana and provided new insights into adaptation to arid environments [25]. Together, these genomic resources offer a useful framework for marker development and genetic characterization in the genus.

Studies of *C. acanthophylla* have mainly focused on ecological distribution, seed germination, seedling cultivation and resource utilization, whereas genetic information across its known distribution in Xinjiang remains limited. Here, whole-genome resequencing data from 33 individuals were used both to develop genome-derived InDel markers and to provide an independent SNP-based assessment of genetic relationships. The resulting InDel markers were then applied to all 93 individuals sampled from 11 field localities. The objectives were to: (1) characterize genome-wide SNP and InDel variation and develop polymorphic PCR-based InDel markers; (2) evaluate genome-wide genetic relationships among the 33 resequenced individuals; (3) determine how well the InDel markers recover the broad genetic patterns observed in the same 33 individuals; (4) use the InDel panel to obtain a preliminary assessment of genetic diversity, regional differentiation and spatial genetic patterns across all 93 individuals; and (5) evaluate the practical value and limitations of the markers for germplasm characterization and conservation-oriented sampling.

## 2. Results

### 2.1. Genome-Wide Discovery and Distribution of Candidate InDel Loci

After quality control of resequencing data from 33 *C. acanthophylla* individuals used for candidate InDel development, a total of 1029.68 Gb of clean bases was obtained. The clean base output per sample ranged from 17.98 to 46.30 Gb, with an average of 31.20 Gb. Q20 values ranged from 97.07% to 98.09%, Q30 values ranged from 92.64% to 95.66%, and GC content ranged from 34.55% to 36.52%. The mapping rate of clean reads to the *C. arborescens* reference genome ranged from 98.19% to 99.76%, the mean sequencing depth ranged from 17× to 44×, and genome coverage ranged from 96.22% to 97.76%. After variant detection and filtering, 2,873,410 high-quality polymorphic SNPs were obtained across the eight chromosomes. These results indicate that the resequencing data were of high quality and provide a reliable basis for candidate InDel screening and molecular marker development in *C. acanthophylla*.

Based on alignment to the *C. arborescens* reference genome, 5,679,915 InDel loci were identified, indicating abundant insertion/deletion variation between *C. acanthophylla* and the reference genome (Table 1). The total length of these InDel loci was 30,018,850 bp, accounting for 2.4687% of the reference genome. In terms of variant type, the number of deletion-type InDels was 3,078,634, higher than the number of insertion-type InDels (2,601,281). However, the total length of insertions was 16,577,695 bp, higher than the total length of deletions (13,441,155 bp), suggesting that insertions were fewer in number but may have had a greater average fragment length.

The number of InDel loci was unevenly distributed among chromosomes. Chromosome 1 contained the largest number of InDels (833,829), followed by chromosomes 3 (809,962), 4 (782,538), 2 (742,946), 5 (726,063), 7 (671,286) and 6 (647,422). Chromosome 8 had the lowest number of InDels (420,394), and 45,475 InDels were detected in scaffold regions. These results indicate that InDel variation is abundant in the *C. acanthophylla* genome and provides sufficient candidate loci for molecular marker development.

### 2.2. Polymorphism Analysis of InDel Primers

A total of 34 candidate InDel primer pairs were initially designed. After preliminary amplification and polymorphism evaluation, eight primer pairs with stable amplification, clear genotyping profiles, and detectable polymorphism were retained for subsequent analyses. The number of alleles (Na) ranged from 1.200 to 2.800, with a mean of 1.950, and the effective number of alleles (Ne) ranged from 1.056 to 2.359 (Table 2). Shannon’s information index (I) ranged from 0.075 to 0.913. Locus 8 showed the highest polymorphism, with Na = 2.800, Ne = 2.359 and I = 0.913. Observed heterozygosity (Ho) ranged from 0.000 to 1.000, whereas expected heterozygosity (He) ranged from 0.044 to 0.561. Ho reached 1.000 at locus 7, while loci 8 and 13 had Ho values of 0.900 and 0.865, respectively. The polymorphism information content (PIC) ranged from 0.0835 to 0.5603, with a mean of 0.3659. Locus 8 had the highest PIC value (0.5603), followed by locus 7 (0.5333), whereas loci 9 and 11 had relatively low PIC values (0.1218 and 0.0835, respectively). These results indicate substantial variation in marker informativeness among the eight loci.

The fixation index (Fis) varied greatly among loci, ranging from −0.9572 to 1.0000, indicating different degrees of heterozygote deficiency or excess among loci. Loci 9 and 11 both had Fis values of 1.0000, indicating obvious heterozygote deficiency, whereas loci 7 and 13 showed strongly negative Fis values, indicating heterozygote excess. After correction for multiple testing, five of the eight loci (7, 8, 11, 12 and 13) showed significant deviation from Hardy–Weinberg equilibrium (HWE) in at least one sampled group, whereas loci 2, 6 and 9 showed no significant deviation. Most loci that deviated from HWE had negative Fis values, suggesting that these deviations were mainly associated with heterozygote excess rather than a general pattern of heterozygote deficiency. Fst values ranged from 0.0608 to 0.8746, indicating substantial variation among loci in their estimates of genetic differentiation. Correspondingly, Nm ranged from 0.0359 to 3.8613, suggesting substantial variation in inferred gene flow among loci. Overall, the eight InDel loci showed variable levels of polymorphism and provided useful preliminary information on genetic diversity and differentiation among the sampled groups.

### 2.3. Genome-Wide SNP-Based Genetic Relationships Among the 33 Resequenced Individuals

**Figure 1:**
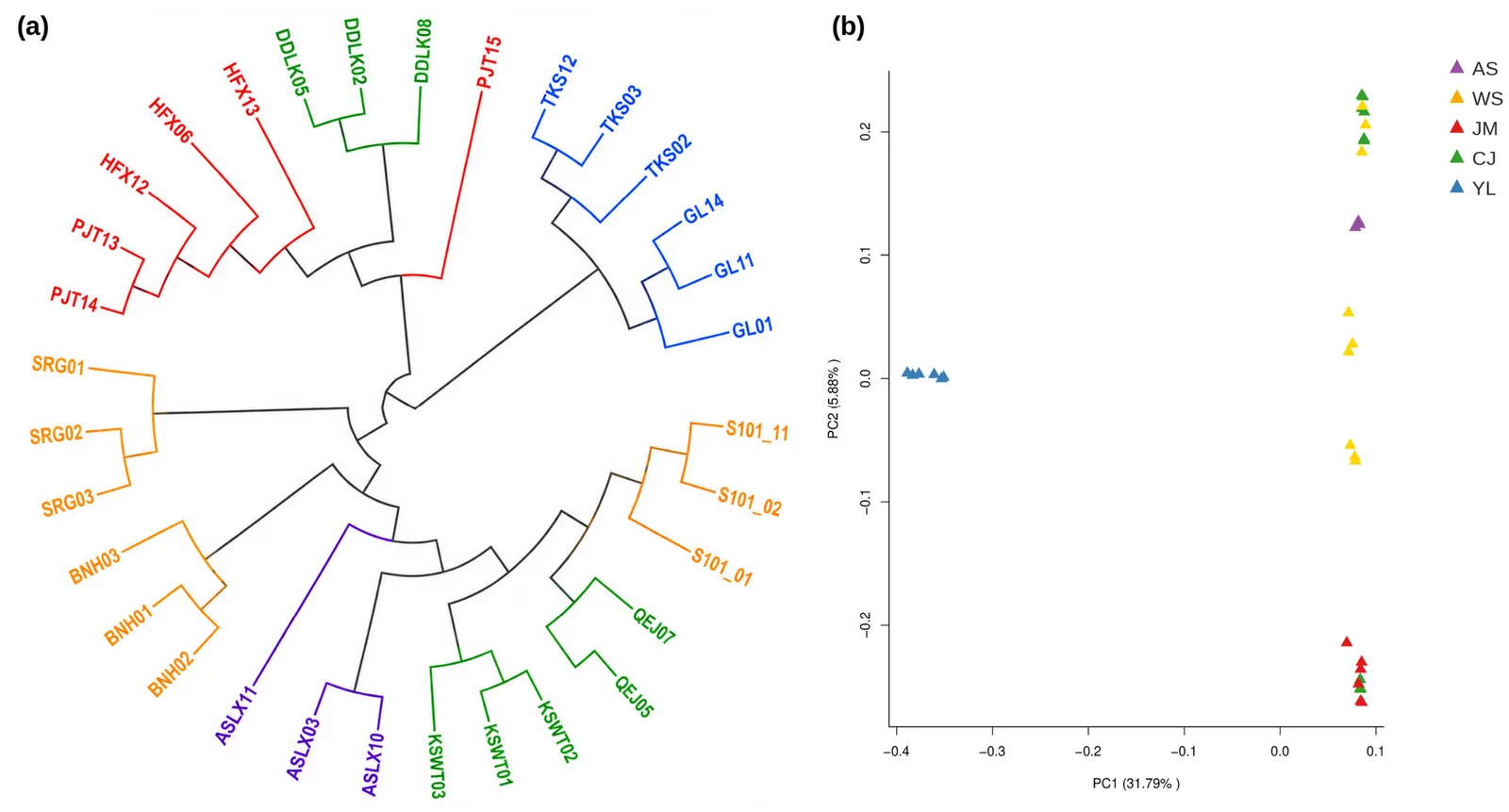
Genome-wide SNP-based genetic relationships among the 33 resequenced C. acanthophylla individuals. (**a**) Neighbor-joining (NJ) tree based on genome-wide SNPs and constructed using TreeBeST v1.9.2. Branch and label colors indicate the five exploratory analytical groups. (**b**) Principal component analysis performed using GCTA v1.91.3. PC1 and PC2 explained 31.79% and 5.88% of the total genomic variation, respectively. Colors indicate the five exploratory analytical groups: AS, WS, JM, CJ, and YL.

**Figure 2:**
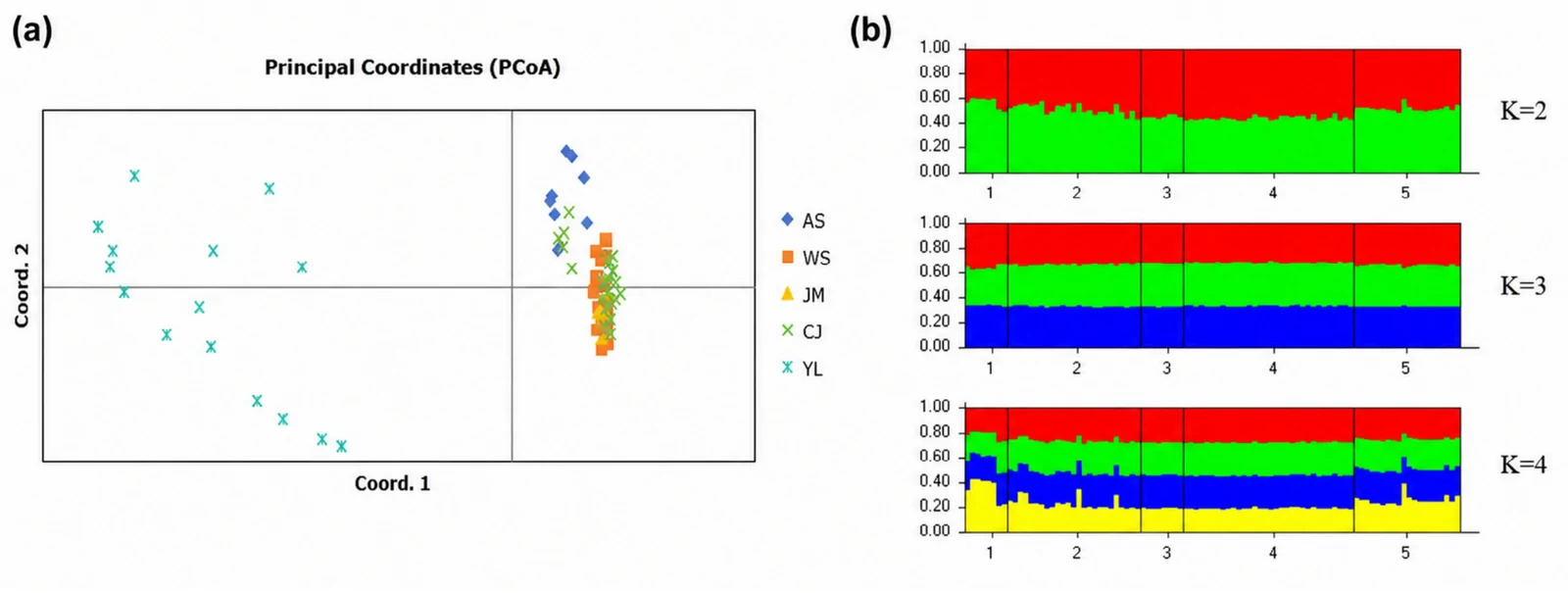
Population genetic relationships and genetic structure of 93 *C. acanthophylla* individuals based on eight InDel markers. (**a**) Principal coordinate analysis (PCoA). Different colors and shapes represent different analytical groups; the first and second coordinate axes explained 58.51% and 7.41% of the genetic variation, respectively. (**b**) STRUCTURE bar plots for K = 2 to K = 4. Each individual is represented by a vertical bar divided into colored segments, indicating the estimated ancestry proportion assigned to each cluster. Numbers below the plots indicate analytical population codes.

Genome-wide SNP analysis of the 33 resequenced individuals revealed clear regional genetic differentiation (Figure 1). In the PCA, PC1 and PC2 explained 31.79% and 5.88% of the total genomic variation, respectively (Figure 2b). YL individuals were clearly separated from the remaining samples along PC1, while additional differentiation occurred among the non-YL materials. The SNP-based NJ tree also showed stronger locality-associated clustering, with several individuals from the same or geographically related sampling localities forming relatively coherent branches (Figure 1a). SNP-based PCA revealed clear genetic differentiation among subsets of the sampled individuals (Figure 1b).

PC1 and PC2 explained 31.79% and 5.88% of the total genomic variation, respectively. YL individuals were clearly separated from the remaining samples along PC1, whereas additional differentiation was observed among the non-YL materials. The SNP-based genetic relationship tree also showed pronounced locality-associated clustering, with individuals from several sampling localities forming relatively coherent branches (Figure 2a).

Analysis of the same 33 individuals using the eight InDel loci recovered part of the broad regional differentiation observed in the SNP dataset, particularly the differentiation of YL, but locality-level relationships were less consistently resolved (Figure S2). Thus, the eight-marker panel captured some major regional differences but provided substantially lower resolution than the genome-wide SNP dataset for fine-scale genetic relationships.

### 2.4. Preliminary Genetic Diversity Assessment of the 93 Individuals Using the Developed InDel Markers

As a preliminary application of the developed markers, genetic diversity was evaluated among the five exploratory analytical groups. Na ranged from 1.308 to 1.769, and Ne ranged from 1.222 to 1.514. Shannon’s information index (I) ranged from 0.185 to 0.402 (Table 3). The AS analytical population had the highest I value (0.402), indicating relatively high genetic diversity, whereas the YL analytical population had the lowest I value (0.185), suggesting relatively low genetic variation. Ho ranged from 0.199 to 0.365, He ranged from 0.127 to 0.259, and uHe ranged from 0.146 to 0.278.

At the overall level, the mean values of Na, Ne, I, Ho, He and uHe were 1.585, 1.425, 0.316, 0.320, 0.210 and 0.222, respectively, indicating that the genetic diversity of the studied materials was moderate to low. In summary, genetic diversity differed among the five analytical groups, with AS showing relatively high genetic diversity and YL showing the lowest genetic diversity.

### 2.5. Preliminary Genetic Relationships Among the 93 Individuals Based on the Developed InDel Markers

PCoA and STRUCTURE provided complementary, although not identical, views of genetic variation among the sampled materials (Figure 2a,b). In the PCoA plot, the first and second coordinate axes explained 58.51% and 7.41% of the total genetic variation, respectively (Figure 2a).

Individuals assigned to the YL analytical population were clearly separated from the other materials and were mainly distributed on the left side of the first coordinate axis. In contrast, individuals from AS, WS, JM and CJ were mainly clustered on the right side of the plot, indicating relatively close genetic relationships or shared genetic components among these four analytical groups. This pattern suggests a marked genetic differentiation between YL and the remaining populations.

STRUCTURE analysis further supported the genetic pattern revealed by PCoA (Figure 2b). Mean LnP(K) was highest at K = 1 and showed no consistent increase with increasing K, whereas ΔK reached its maximum at K = 2, with a lower secondary peak at K = 3 (Figure S3). Because the Evanno ΔK method does not evaluate K = 1, K = 2 was interpreted as the strongest hierarchical subdivision detected among K ≥ 2 rather than as definitive evidence for two discrete genetic populations. At K = 2, YL showed a relatively distinct ancestry pattern compared with AS, WS, JM, and CJ, whereas the latter four units displayed more similar ancestry proportions. When K increased to 3 and 4, additional genetic components appeared within the non-YL materials, but these patterns were less clearly resolved. Although a secondary ΔK peak occurred at K = 3, it was substantially lower than that at K = 2.

Taken together, the analyses suggest that YL is relatively differentiated from the remaining sampled materials, but the extensive overlap in PCoA and admixture in STRUCTURE indicate that the present eight-locus dataset does not support a sharp division into discrete genetic populations. This differentiation pattern is broadly consistent with geographic distribution, suggesting that geographic distance, discontinuous habitats and regional environmental heterogeneity may have jointly shaped the population genetic structure of *C. acanthophylla*.

### 2.6. UPGMA Cluster Tree Analysis

**Figure 3:**
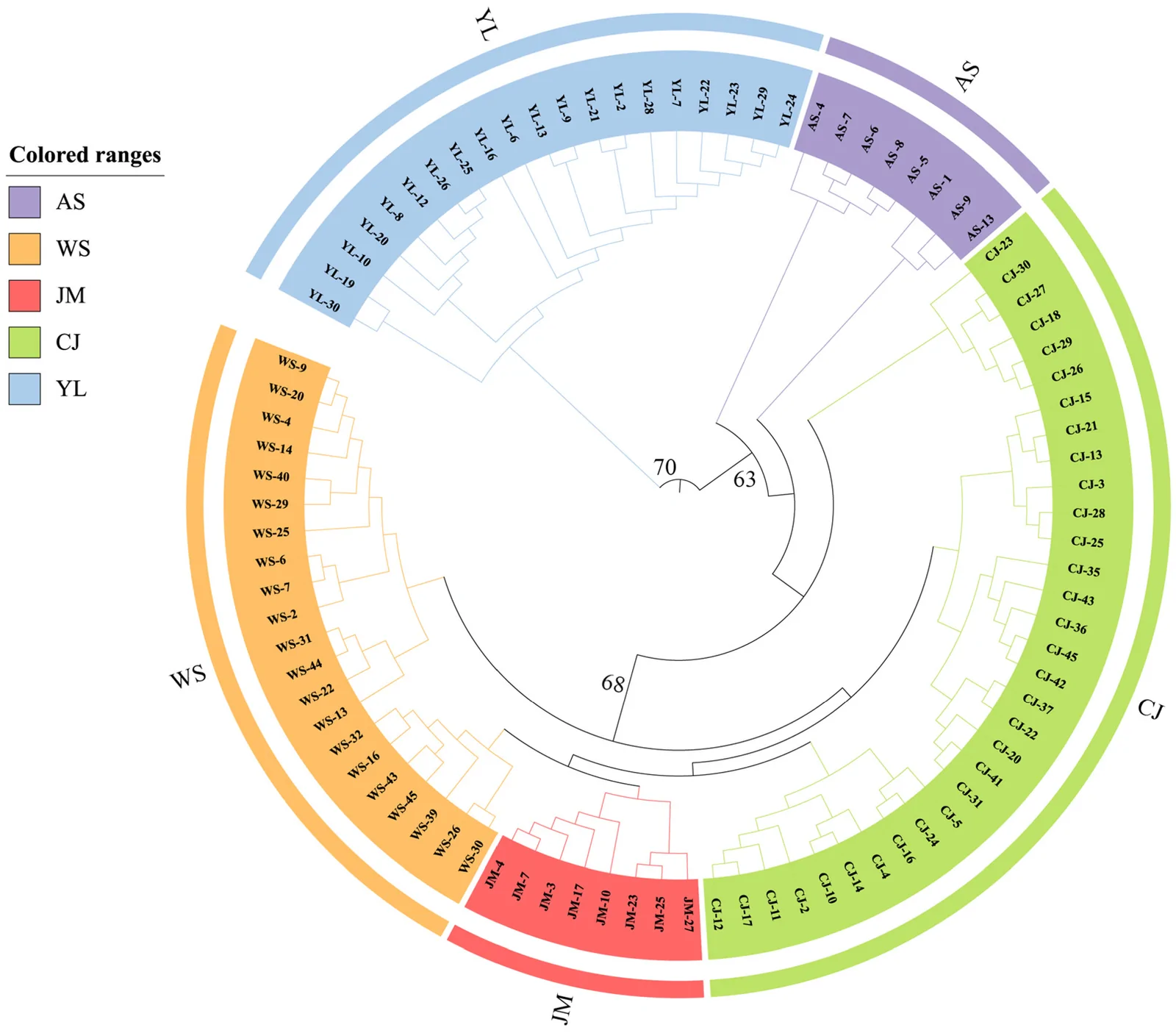
UPGMA tree of 93 *C. acanthophylla* individuals based on eight InDel markers. Branch support was evaluated using 1000 bootstrap replicates. Bootstrap support values ≥ 50% are shown at the corresponding nodes, whereas values < 50% are not displayed. Colored ranges indicate different analytical groups; branch clustering reflects genetic distance and genetic similarity among individuals.

The UPGMA tree constructed from the eight InDel markers showed that the 93 *C. acanthophylla* individuals generally clustered according to genetic distance and geographic origin (Figure 3).

Based on the combined evidence from UPGMA clustering, PCoA, STRUCTURE, genetic distance and geographic continuity, the original 11 field sampling localities were summarized into five analytical groups for downstream population-level interpretation: TKS and GL were assigned to YL; KSW and QEG were assigned to WS; BNH, SRG and S101 were assigned to CJ; PJT, HFX and DLK were assigned to JM; and AS was retained as an independent AS population. Individuals from YL formed a relatively independent major branch and were clearly separated from the other materials, indicating relatively high genetic distinctiveness. This result was largely consistent with the PCoA result, in which YL was located on one side of the first coordinate axis, and with the STRUCTURE result showing two main genetic components.

Most individuals from WS and CJ clustered in relatively concentrated branches, indicating strong genetic similarity within these analytical groups. Individuals from JM were mainly located between the WS and CJ branches and showed some overlap with neighboring groups, suggesting that JM may share genetic backgrounds with adjacent populations or may have experienced a certain degree of gene exchange. Although the AS locality is geographically close to the CJ localities, AS individuals tended to form a separate subcluster adjacent to the CJ branch rather than being fully nested within CJ. In contrast, individuals from BNH, SRG and S101 clustered more closely together and were therefore grouped as the CJ population. This pattern suggests that the genetic composition of AS may differ from that of CJ and supports retaining AS as a separate exploratory analytical group. Overall, the UPGMA analysis illustrated genetic relationships among the sampled materials and showed a marker-based pattern broadly associated with geographic origin.

### 2.7. AMOVA Revealed Marked Differentiation Among Sampling Localities and Analytical Groups

AMOVA based on the 11 original field sampling localities showed that 67.00% of the total molecular variation occurred among localities and 33.00% occurred within localities (PhiPT = 0.669, *p* = 0.001; Table 4). A second AMOVA based on the five exploratory analytical groups showed a highly similar pattern, with 68.37% of the variation occurring among groups and 31.63% within groups. The consistency between the two grouping schemes indicates that the high among-locality component of genetic variation was also evident when the original field sampling units were analyzed independently of the cluster-derived five-group framework.

The high proportion of among-locality variation observed in this study indicates substantial spatial genetic differentiation in *C. acanthophylla*. This pattern may be associated with its discontinuous distribution and the complex mountain–basin landscape of Xinjiang, which may reduce connectivity among sampling localities. Genetic drift may further contribute to the accumulation of genetic differences among geographically isolated localities [15,16,26,27].

### 2.8. Relationship Between Geographic and Genetic Distances

**Figure 4:**
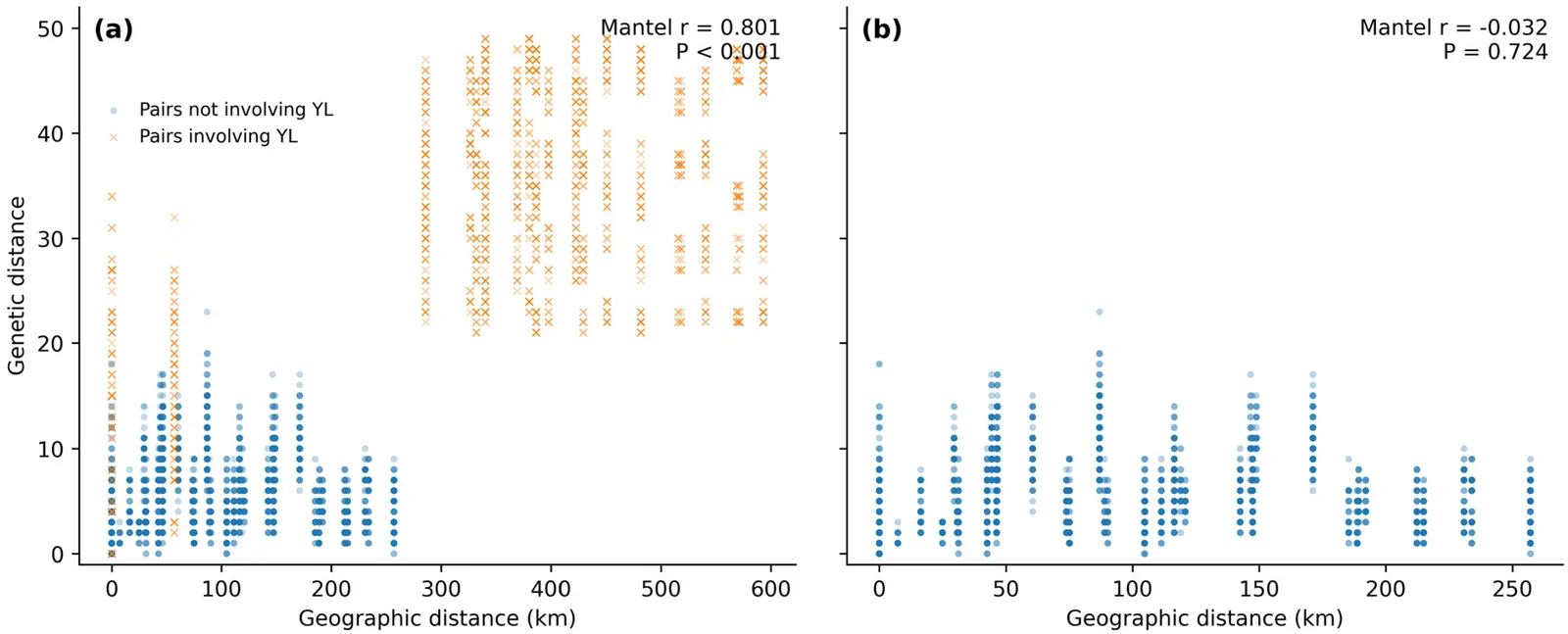
Relationship between geographic and genetic distances in *C. acanthophylla*. (**a**) Pairwise comparisons among all 93 individuals. Comparisons involving YL are distinguished from those not involving YL to illustrate the contribution of the geographically isolated YL group to the overall pattern. (**b**) Pairwise comparisons among the 73 individuals after excluding YL. Mantel tests were performed using 9999 permutations.

The scatter plot of geographic distance and genetic distance showed an evident positive relationship (Figure 4).

The Mantel test based on all 93 individuals showed a strong positive association between genetic and geographic distances (r = 0.801, *p* < 0.001; Figure 4a). However, pairwise comparisons involving YL formed a distinct group at larger geographic distances. To evaluate the influence of YL on the overall relationship, the Mantel test was repeated after excluding YL. No significant association was detected among the remaining 73 individuals (r = −0.032, *p* = 0.724; Figure 4b). These results indicate that the strong overall association between genetic and geographic distances was largely driven by comparisons involving the geographically isolated YL group, rather than by a general isolation-by-distance pattern among the remaining samples.

## 3. Discussion

### 3.1. Development and Application Value of InDel Markers

Whole-genome resequencing identified extensive SNP and InDel variation, providing a basis for both genome-wide assessment and PCR-based marker development in *C. acanthophylla*. Of 34 candidate InDel primer pairs, eight loci with stable amplification, clear genotype profiles and detectable polymorphism were retained. PCR-based InDel markers are inexpensive and readily applicable to larger sample sets [21,22,23], but their resolving power depends on marker number and genomic coverage. Because genome-wide SNP data were available for the same 33 resequenced individuals, the performance of the eight-locus panel could be evaluated directly against a much denser marker dataset.

Comparison of the same 33 individuals showed that the eight InDel markers recovered part of the broad regional pattern detected by genome-wide SNPs, particularly the differentiation of YL, but did not consistently reproduce the finer locality-level relationships resolved by the SNP dataset. This difference is expected given the large disparity in marker density. The comparison therefore clarifies the appropriate role of the developed panel: the eight InDel markers are suitable for low-cost preliminary germplasm characterization and broader sample screening, whereas genome-wide SNP data provide substantially greater resolution for population-level inference.

### 3.2. Genetic Diversity of C. acanthophylla and Its Possible Causes

Across the five exploratory analytical groups, mean Na, Ne, I, Ho, He and uHe were 1.585, 1.425, 0.316, 0.320, 0.210 and 0.222, respectively, indicating moderate to low marker-level genetic diversity. Previous reproductive studies showed that *C. acanthophylla* is highly self-incompatible and obligately outcrossing [14], a mating system that can help maintain genetic variation [12,13]. However, the relatively low He and I values observed here may reflect the discontinuous distribution of the species in Xinjiang, limited effective population size within habitat patches, habitat fragmentation, or restricted effective pollen- and seed-mediated connectivity. Similar factors have been reported to influence genetic variation in Caragana and other arid-region plants [17,18,28]. Because the present inference is based on only eight InDel loci, these mechanisms should be regarded as plausible explanations rather than demonstrated causes.

Genetic diversity differed among exploratory analytical groups. AS showed the highest I, He and uHe values, whereas YL showed the lowest values. AS is located near the middle section of the northern Tianshan slope, where heterogeneous foothill, valley and desert-steppe habitats occur in close proximity. Such habitat heterogeneity may contribute to the observed diversity pattern, but environmental variables were not tested directly in this study. AS was also genetically distinguishable from nearby CJ in the InDel-based analyses, indicating that geographic proximity alone does not fully explain their relationship. Possible contributions from local demographic history, habitat connectivity or environmental heterogeneity remain to be tested with denser genomic and environmental data [29,30].

YL showed the lowest InDel-based genetic diversity but the clearest regional differentiation. Its geographic isolation in the Yili Valley and adjacent western Tianshan region may have contributed to this pattern, although demographic history and genetic drift were not directly evaluated. Importantly, YL differentiation was also evident in the genome-wide SNP analysis of the resequenced subset. This cross-marker agreement supports YL as a distinct regional pattern in the present dataset, while broader within-locality genome-wide sampling is still required for robust population-level inference.

Some loci and exploratory analytical groups showed Ho values higher than He and negative fixation indices, indicating heterozygote excess in part of the InDel dataset. These patterns may reflect locus-specific variation, unequal sample sizes among original sampling localities, within-group genetic heterogeneity, or the limited number of loci. Because systematic replicate genotyping was not performed, technical reproducibility could not be quantified independently. Heterozygosity and fixation-index estimates should therefore be interpreted cautiously and should not be used alone to infer mating-system effects or the genetic status of individual groups.

### 3.3. Population Genetic Differentiation Under Geographic and Environmental Heterogeneity

AMOVA attributed 68.37% of the total InDel variation to differences among the five exploratory analytical groups, while an independent analysis of the 11 original sampling localities produced a similar among-locality component (67.00%). PCoA, STRUCTURE and UPGMA all distinguished YL from the other materials. The Mantel test detected a strong association between genetic and geographic distances across all 93 individuals (r = 0.801, *p* < 0.001), but this relationship disappeared after YL was excluded (r = −0.032, *p* = 0.724). Thus, the overall spatial signal was driven largely by comparisons involving the geographically isolated YL group rather than by a general isolation-by-distance pattern among the remaining samples.

The geographic position of YL provides a plausible context for its differentiation. TKS and GL occur in the western Yili Valley region, whereas most other localities lie along the middle-eastern northern Tianshan slope and the southern margin of the Junggar Basin. The approximately 300 km distribution gap and the absence of continuous populations in the intervening area may reduce connectivity between YL and the other sampled materials. The broader mountain–basin setting of Xinjiang is also characterized by marked climatic and hydrological heterogeneity [31]. Regional phylogeographic studies have reported distinct historical patterns among plant populations in the Yili Valley, western Junggar and Tianshan regions [28,32,33]. These studies provide regional context but do not directly establish the historical origin of YL differentiation.

YL differentiation was supported at both marker scales: it was evident in the 93-individual InDel analyses and in the genome-wide SNP analysis of the 33 resequenced individuals. Nevertheless, the two datasets have complementary limitations. The SNP dataset offers high genomic resolution but includes only three individuals per locality, whereas the InDel dataset provides broader individual sampling but is based on only eight loci. In addition, polymorphism was considered during InDel marker screening, which may bias the panel toward more variable loci. Robust population delimitation will therefore require genome-wide data with broader within-locality sampling.

Among the remaining exploratory groups, geographic distance alone did not explain all observed relationships. WS, CJ and JM were relatively close geographically and shared more genetic similarity in the InDel-based analyses. AS, although geographically near CJ, remained partially differentiated. These patterns may reflect shared ancestry, historical connectivity or local demographic differences, but the present marker set cannot resolve these mechanisms. Environmental explanations are likewise tentative because climatic, soil and habitat variables were not incorporated into the genetic analyses.

The partial differentiation between AS and CJ illustrates that geographic proximity does not necessarily correspond to genetic similarity. Similar patterns have been reported in *C. microphylla*, where geographic, environmental and historical factors jointly influence population structure [17,18]. In *C. acanthophylla*, however, distinguishing among these mechanisms will require explicit environmental analyses and denser genome-wide sampling [34].

### 3.4. Implications for Germplasm Conservation and Seed-Source Management

The present results provide a preliminary basis for germplasm conservation and utilization in *C. acanthophylla*. Conservation sampling should consider both genetic diversity and regional differentiation. AS retained relatively high InDel-based diversity, whereas YL showed the clearest differentiation across both InDel and SNP datasets. These contrasting patterns suggest that both regions merit representation in future germplasm collections and conservation assessments. For arid-region shrubs with discontinuous distributions, maintaining both diverse and differentiated materials can help preserve overall genetic variation and evolutionary potential [27,35,36,37].

The five exploratory analytical groups may serve as a provisional framework for regional sampling and provenance documentation, but they should not be treated as formally defined conservation or seed-source units. The distinct pattern of YL supports ensuring its representation in ex situ collections, while the differentiation observed in AS highlights the importance of maintaining clear provenance records. Formal management-unit designation will require denser genome-wide markers and broader within-locality sampling.

### 3.5. Application Prospects and Limitations

The primary contribution of this study is the development and experimental validation of genome-derived InDel markers for *C. acanthophylla* and the evaluation of their performance against genome-wide SNP patterns. The SNP dataset provided a high-resolution assessment of the 33 resequenced individuals, whereas the InDel panel extended genetic screening to all 93 individuals. These datasets are complementary rather than equivalent: SNPs provide much greater genomic resolution but limited within-locality sampling depth, while the InDel panel offers broader individual coverage at low genotyping cost but limited genomic representation. A further limitation is that systematic replicate PCR or independent genotyping was not performed to quantify technical reproducibility. Accordingly, the 93-individual InDel analyses should be regarded as preliminary germplasm characterization. Future population-genomic studies should combine dense genome-wide markers with broader within-locality sampling before formal population, conservation or seed-source units are delimited.

## 4. Materials and Methods

### 4.1. Plant Materials

**Figure 5:**
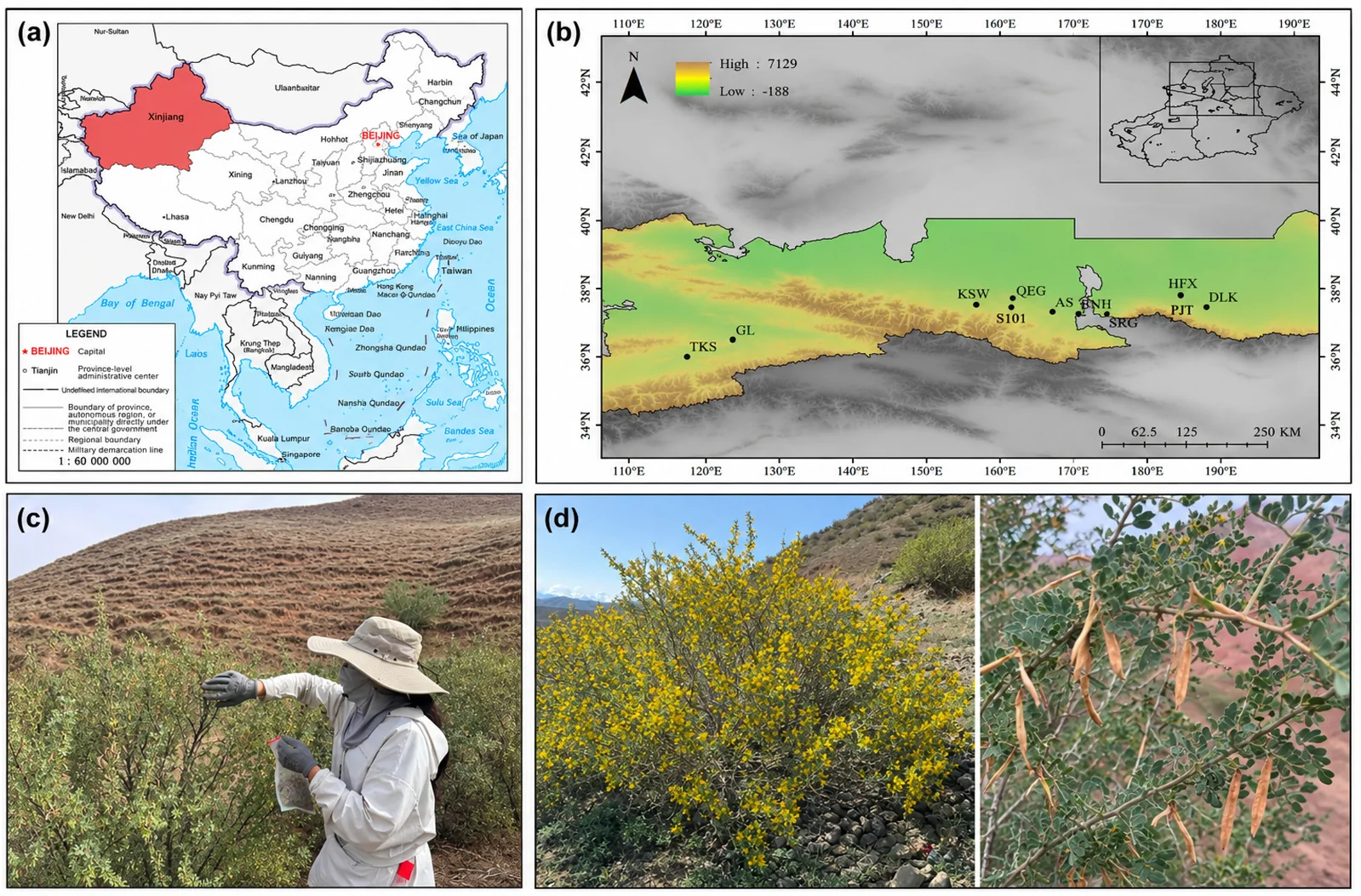
Geographic location, sampling sites, and representative morphology of *C. acanthophylla*. (**a**) Location of Xinjiang in China. (Map approval number: GS(2019)1652) (**b**) Distribution map of the original 11 field sampling localities of *C. acanthophylla* in Xinjiang, China. Filled circles indicate sampling localities, and locality codes are shown next to the sampling points. The inset in the upper right shows the relative positions of the sampling sites within Xinjiang. (**c**) Field collection of *C. acanthophylla* individuals from natural populations. (**d**) Representative habitat and morphological characteristics of *C. acanthophylla*, showing a flowering shrub in its natural habitat and fruiting branches with mature pods.

Field surveys were conducted at 11 localities within the known distribution range of *Caragana acanthophylla* in Xinjiang, China: TKS, GL, AS, KSW, QEG, BNH, SRG, S101, PJT, HFX, and DLK. Before sampling, the habitat conditions at each locality were surveyed, and the geographic coordinates, elevation, and habitat type were recorded. A total of 93 naturally occurring individuals were sampled. All individuals were identified as *C. acanthophylla* according to the taxonomic descriptions and diagnostic morphological characteristics provided in *Flora of China* and *Flora Xinjiangensis* [11,38]. Only healthy, mature individuals without obvious disease, insect damage, or severe mechanical injury were selected. To reduce the likelihood of repeatedly sampling closely related individuals, the distance between sampled individuals within each locality was maintained at greater than 10 m. The distance between different field localities was greater than 10 km. Each sampled individual was assigned a unique code, and its sampling locality and associated field information were recorded. Fresh, healthy young leaves were collected separately from each individual, immediately placed in individually labelled sampling bags containing silica gel, rapidly dried, and transported to the laboratory for DNA extraction. Representative individuals and their habitats were photographed in the field. All 11 localities were retained as the original field sampling units and are shown on the distribution map (Figure 5). Detailed information on the 11 original sampling localities, including locality names, geographic coordinates, elevation, sample sizes, and their corresponding exploratory analytical groups, is provided in Table S1.

Spatially, TKS and GL are located in the Yili Valley and adjacent mountains of western Xinjiang, whereas the remaining localities are broadly distributed along the middle-eastern section of the northern Tianshan slope and the southern margin of the Junggar Basin. No specific collection permit was required for this species at the sampled localities; however, sampling was conducted with the permission of the relevant local management authorities.

### 4.2. DNA Extraction and Quality Assessment

Genomic DNA was extracted from 93 *C. acanthophylla* individuals using a polysaccharide- and polyphenol-rich plant genomic DNA extraction kit (DP360, Tiangen Biotech, Beijing, China). DNA purity was evaluated using a NanoDrop One microvolume UV–Vis spectrophotometer (Thermo Fisher Scientific, Waltham, MA, USA),and DNA integrity was assessed by agarose gel electrophoresis. DNA samples with sufficient concentration, acceptable purity, and intact, non-degraded DNA bands were diluted to the required concentration and stored at −20 °C until use. All 93 individuals were used for InDel genotyping and population genetic analyses. For whole-genome resequencing, three individuals were randomly selected from each of the 11 field localities. All selected samples met the DNA quality requirements for library construction, resulting in a total of 33 individuals.

### 4.3. Screening of InDel Loci and Primer Development

The chromosome-level nuclear genome of *Caragana arborescens* published by Cui et al. [24] was used as the reference genome. The assembly was obtained from the NCBI database (BioProject PRJNA978347; GenBank assembly accession GCA_051861645.1; assembly name ASM5186164v1). The 33 selected *C. acanthophylla* individuals were subjected to whole-genome resequencing. Short-insert genomic DNA libraries with an average insert size of 250-350 bp were constructed using the QuarPrep EZ DNA Library Preparation Kit (Shanghai Diyan Biotechnology Co., Ltd., Shanghai, China; catalog no. NL1012) and sequenced on the DNBSEQ-T7 platform. Raw reads were quality-filtered by removing reads containing adaptor sequences, reads with more than 10% N bases, and low-quality reads in which bases with quality scores below 10 accounted for more than 50% of the total read length. Clean reads were aligned to the *C. arborescens* reference genome using BWA. The resulting alignments were preprocessed by duplicate removal, local realignment around InDel regions, and base-quality score recalibration, and SNPs and InDels were subsequently called and filtered using GATK to retain high-confidence variants. Candidate InDel loci were selected from the resulting variant dataset based on InDel length, chromosomal distribution, conserved flanking sequences, and expected allele-size differences suitable for agarose-gel discrimination. Thirty-four candidate loci were screened by PCR. Primer pairs were retained when they produced stable amplification, clear bands within the expected size range, no obvious nonspecific amplification or severe smearing, unambiguous genotype profiles, and detectable polymorphism. No fixed threshold for PIC, heterozygosity, or allele number was applied. Finally, eight InDel loci were retained for genotyping all 93 individuals (Table S2). Ambiguous or unscorable amplification products were recorded as missing data.

### 4.4. PCR Amplification and Genotype Scoring

PCR amplification was performed in a 25 μL reaction mixture using a VeritiPro 96-Well Thermal Cycler (Applied Biosystems, Foster City, CA, USA), containing 1 μL template DNA, 1 μL forward primer, 1 μL reverse primer, 12 μL 2× EasyTaq PCR SuperMix (+dye) and 10 μL deionized water.

The PCR amplification program was as follows: initial denaturation at 94 °C for 4 min and 30 cycles of denaturation at 94 °C for 30 s, annealing at 55 °C for 35 s and extension at 72 °C for 30 s, followed by a final extension at 72 °C for 10 min and storage at 4 °C.

PCR products were separated by 2% agarose gel electrophoresis using a DYCP-31DN horizontal electrophoresis system (Beijing Liuyi Biotechnology Co., Ltd., Beijing, China), and genotypes were scored according to amplicon size. For each InDel locus, the shorter and longer fragments were designated as alleles 0 and 1, respectively. Individuals showing a single band corresponding to the shorter or longer allele were scored as homozygotes (0/0 or 1/1), whereas individuals showing both bands were scored as heterozygotes (0/1). A representative electrophoretic profile illustrating genotype scoring is shown in Figure S1. The resulting genotypes were recorded in diploid format for subsequent analyses [21,22,23]. Systematic replicate PCR or independent genotyping of a predefined subset of samples was not performed in the present study.

### 4.5. Genetic Diversity and Population Structure Analyses

Genetic diversity parameters were calculated for loci and analytical groups using GenAlEx 6.5, including the number of alleles (Na), effective number of alleles (Ne), observed heterozygosity (Ho), expected heterozygosity (He), unbiased expected heterozygosity (uHe), Shannon’s information index (I), gene flow (Nm), inbreeding coefficient (Fis) and fixation index (Fst). The 11 original sampling localities were retained as the primary field sampling units. Exploratory UPGMA clustering, together with PCoA, STRUCTURE, genetic distance, and geographic information, was used to summarize the 11 sampling localities into five analytical groups (YL, AS, WS, CJ, and JM) for regional comparison. Because these groups were partly derived from the same marker data used in subsequent population-level analyses, statistics based on the five-group scheme were treated as exploratory rather than as independent evidence of population delimitation. To reduce the risk of circular inference, an additional AMOVA was performed using the 11 original sampling localities as predefined groups, independent of the five cluster-derived analytical groups. GenAlEx supports analyses of diploid codominant markers, binary markers, haplotypes and DNA sequences, and can be used for heterozygosity estimation, AMOVA, PCoA and Mantel tests [39].

Principal coordinate analysis (PCoA) was performed in GenAlEx 6.5 based on the genetic distance matrix to visualize genetic relationships among individuals and analytical groups. Analysis of molecular variance (AMOVA) was also conducted in GenAlEx 6.5 to quantify the distribution of genetic variation within and among populations, and the statistical significance of the variance components was evaluated using 999 permutations. The genetic distance matrix was imported into MEGA 11.0, and the unweighted pair-group method with arithmetic mean (UPGMA) was used to construct a genetic relationship tree [40,41]. The UPGMA tree was used mainly to assist in interpreting genetic relationships and clustering patterns among materials from different geographic origins. In addition, a geographic distance matrix was calculated from the coordinates of sampled individuals, and a Mantel test was performed in GenAlEx 6.5 to assess the correlation between the genetic and geographic distance matrices using 9999 permutations. The Mantel correlation coefficient (r) and permutation-based P value were reported. Geographic distances were calculated from the coordinates of the sampling localities. Mantel tests with 9999 permutations were used to evaluate the association between genetic and geographic distance matrices. The analysis was first performed using all 93 individuals and was subsequently repeated after excluding the geographically isolated YL group to examine the robustness of the observed association. Mantel correlation coefficients (r) and permutation-based P values were reported.

Population genetic structure was inferred using STRUCTURE 2.3 [42]. The admixture ancestry model with correlated allele frequencies was used, and no prior population information was incorporated into the analysis. The number of clusters (K) was set from 1 to 10, and each K value was run independently 15 times. Each run used a burn-in period of 100,000 iterations followed by 100,000 Markov chain Monte Carlo (MCMC) iterations. Mean LnP(K) and the Evanno ΔK statistic were both examined to evaluate clustering patterns [43], and STRUCTURE HARVESTER was used to summarize and visualize the STRUCTURE output [44]. Because ΔK cannot be calculated for K = 1, K = 2 was interpreted as the strongest hierarchical subdivision detected among K ≥ 2 rather than as definitive evidence for two discrete populations.

### 4.6. Genome-Wide SNP-Based Genetic Analysis

To provide an independent genome-wide assessment of genetic relationships among the 33 resequenced individuals, the 2,873,410 high-quality SNPs retained after variant filtering were used for subsequent analyses. Principal component analysis (PCA) was performed using GCTA v1.91.3 [45] to summarize genome-wide genetic variation among individuals. A neighbor-joining (NJ) tree based on genome-wide SNPs was constructed using TreeBeST v1.9.2 [46] to evaluate genetic relationships and clustering patterns among the resequenced materials. To directly assess correspondence between the genome-wide SNP dataset and the developed InDel markers, the same 33 individuals were extracted from the eight-InDel dataset and analyzed separately. The two marker systems were compared qualitatively with respect to broad regional differentiation and locality-level resolution.

## 5. Conclusions

Whole-genome resequencing of 33 *C. acanthophylla* individuals identified 2,873,410 high-quality SNPs and 5,679,915 InDels, providing a genomic resource for both genetic assessment and molecular marker development. Eight polymorphic InDel loci with stable amplification and clear genotyping profiles were retained from 34 candidate primer pairs and applied to 93 individuals from 11 original sampling localities. Genome-wide SNP analyses revealed clear regional differentiation and finer locality-associated genetic relationships. In the same 33 individuals, the eight InDel loci recovered part of the broad regional pattern, particularly the differentiation of YL, but showed lower resolution for fine-scale relationships. Across all 93 individuals, the InDel panel revealed moderate to low marker-level genetic diversity and detectable regional differentiation. AS retained comparatively high genetic diversity, whereas YL showed the lowest diversity but the clearest genetic distinctiveness. AMOVA based on the 11 original sampling localities and the five exploratory analytical groups produced similar patterns, with most genetic variation occurring among sampling units. The overall association between genetic and geographic distances was largely driven by the geographic separation of YL and was no longer significant after YL was excluded. These findings support representative collection of differentiated YL materials and careful provenance documentation of AS and other regional germplasm. The developed InDel panel therefore provides a practical and low-cost tool for preliminary germplasm characterization, provenance screening and broader individual sampling. However, the five exploratory analytical groups should not be regarded as formally delimited biological populations, conservation units or seed-source units. Future studies should combine genome-wide markers with broader within-locality sampling and independent technical replication.

## Figures and Tables

**Table 1:** Distribution characteristics of InDel loci obtained by alignment to the *C. arborescens* reference genome.

Genome Size (Mb): 1,215.95	Chr.	Number of InDels
Total number of InDels: 5,679,915	1	833,829
Total length of InDels (bp): 30,018,850	2	742,946
Insertion count: 2,601,281	3	809,962
Insertion total length (bp): 16,577,695	4	782,538
Deletion count: 3,078,634	5	726,063
Deletion total length (bp): 13,441,155	6	647,422
Frequency (InDels/Mb): 4671.17	7	671,286
Density (bp/Mb): 24,687.56	8	420,394
Total content of genome InDels (%): 2.4687	scaffolds	45,475

**Table 2:** Genetic diversity parameters of the eight InDel loci.

**Locus**	Na	Ne	I	Ho	He	PIC	Fis	Fst	Nm	HWE
2	2.200	1.626	0.504	0.334	0.306	0.4871	−0.0900	0.3873	0.3954	NS
6	2.000	1.655	0.516	0.461	0.352	0.3853	−0.3100	0.2701	0.6756	NS
7	2.200	2.049	0.731	1.000	0.511	0.5333	−0.9572	0.1335	1.6220	D
8	2.800	2.359	0.913	0.900	0.561	0.5603	−0.6054	0.1578	1.3341	D
9	1.200	1.056	0.075	0.000	0.044	0.1218	1.0000	0.8746	0.0359	NS
11	1.200	1.176	0.132	0.000	0.094	0.0835	1.0000	0.5714	0.1875	D
12	2.000	1.713	0.583	0.606	0.399	0.3815	−0.5173	0.1573	1.3391	D
13	2.000	1.895	0.656	0.865	0.465	0.3745	−0.8589	0.0608	3.8613	D

Note: Na = mean number of alleles; Ne = effective number of alleles; I = Shannon’s information index; Ho = observed heterozygosity; He = expected heterozygosity; PIC, polymorphism information content; higher PIC values indicate greater marker informativeness; Fis = inbreeding coefficient; Fst = fixation index; Nm = indirect Fst-derived estimate under equilibrium assumptions; HWE, Hardy–Weinberg equilibrium; D, significant deviation from HWE in at least one analytical group after multiple-testing correction; NS, no significant deviation detected.

**Table 3:** Genetic diversity parameters of the five analytical groups of *C. acanthophylla*.

Pop		Na	Ne	I	Ho	He	uHe	F
AS	Mean	1.769	1.514	0.402	0.327	0.259	0.278	−0.144
	SE	0.201	0.155	0.105	0.119	0.067	0.072	0.217
WS	Mean	1.692	1.488	0.353	0.360	0.236	0.241	−0.517
	SE	0.237	0.155	0.111	0.123	0.074	0.075	0.142
JM	Mean	1.538	1.446	0.305	0.365	0.204	0.218	−0.796
	SE	0.215	0.169	0.114	0.134	0.075	0.080	0.038
CJ	Mean	1.615	1.456	0.336	0.351	0.224	0.228	−0.548
	SE	0.213	0.153	0.109	0.118	0.072	0.073	0.096
YL	Mean	1.308	1.222	0.185	0.199	0.127	0.146	−0.524
	SE	0.133	0.101	0.081	0.093	0.056	0.064	0.092
Total	Mean	1.585	1.425	0.316	0.320	0.210	0.222	−0.469
	SE	0.090	0.066	0.046	0.052	0.030	0.032	0.066

Note: Na = number of alleles; Ne = effective number of alleles; I = Shannon’s information index; Ho = observed heterozygosity; He = expected heterozygosity; uHe = unbiased expected heterozygosity; F = fixation index; SE = standard error of the mean.

**Table 4:** Analysis of molecular variance (AMOVA) based on the 11 original sampling localities and the five exploratory analytical groups of *C. acanthophylla.*

Grouping Scheme	Source of Variation	df	SS	MS	Estimated Variance	Variation (%)
11 original sampling localities	Among populations	10	528.982	52.898	5.976	67.00
	Within populations	82	242.620	2.959	2.959	33.00
	Total	92	771.602		8.935	100.00
Five exploratory analytical groups	Among populations	4	491.510	122.877	6.8798	68.37
	Within populations	88	280.093	3.183	3.1829	31.63
	Total	92	771.602		10.0627	100.00

Note: df = degrees of freedom; SS = sum of squares; MS = mean square. For the AMOVA based on the 11 original sampling localities, PhiPT = 0.669 and P = 0.001 based on 9999 permutations. The five exploratory analytical groups were partly derived from genotype-based clustering and were used as a secondary analytical framework rather than as formally delimited biological populations.

## Data Availability

The reference genome of *C*. *arborescens* was obtained from the NCBI database (assembly ASM5186164v1; GenBank assembly accession GCA_051861645.1). The raw whole-genome resequencing data generated for the 33 *C. acanthophylla* individuals are publicly available in the Genome Sequence Archive at the National Genomics Data Center, China National Center for Bioinformation, under BioProject accession PRJCA068505 and GSA accession CRA048374. Additional data supporting the findings of this study are available from the corresponding author upon reasonable request.

## Supplementary Materials

The following supporting information can be downloaded at: https://www.mdpi.com/article/10.3390/plants15172733/s1. Table S1: Information on the 11 original sampling localities; Table S2: Information for the eight retained polymorphic InDel primer pairs; Figure S1: Representative agarose gel electrophoresis profile for InDel genotype scoring; Figure S2: UPGMA tree of the same 33 resequenced individuals based on the eight developed InDel markers; Figure S3: Mean LnP(K) and ΔK results for STRUCTURE analyses.
