# Supplementary material for: Development of Genome-Derived InDel Markers and Genetic Diversity Analysis of Caragana acanthophylla in Xinjiang, China
Source: Plants (Basel). 2026 Sep 7;15(17):2733. doi: 10.3390/plants15172733 (PMC13567721; doi:10.3390/plants15172733)
Supplement: Supplementary file 1 [file plants-15-02733-s001.zip › plants-4483115-supplementary.pdf]

Table S1. Sampling information for the 11 original field localities of *C.acanthophylla* in Xinjiang, China

Locality code	Full locality name	Longitude (°E)	Latitude (°N)	Elevation (m)	Sample size (N)	Exploratory group
AS	Ashili Kazak Township, Changji City	87°02′35.1678″	43°51′11.8724″	958	7	AS
BNH	Bainiaohu, Urumqi City	87°24′24.2833″	43°48′45.0678″	951	8	CJ
SRG	Shirengou, Urumqi City	87°47′47.0132″	43°48′56.9647″	1026	10	CJ
S101	Keziletasi Village, Hutubi County	86°31′47.6204″	44°03′06.2930″	746	4	CJ
PJT	Panjiataizi Village, Jimsar County	88°53′23.3610″	44°00′38.8469″	959	4	JM
HFX	Santai Town, Jimsar County	88°50′58.6885″	44°04′11.5171″	722	10	JM
DLK	Dalongkou Village, Jimsar County	89°10′39.3005″	43°55′29.7996″	949	14	JM
KSW	Kensiwate Reservoir, Manas County	85°58′00.4862″	43°57′16.2711″	889	8	WS
QEG	Qiaoergou Town, Hutubi County	86°29′40.1638″	43°54′23.9176″	1034	8	WS
GL	Gongliu County, Yili Kazak Autonomous Prefecture	82°29′55.9896″	43°23′41.3952″	892	12	YL
TKS	Tekes County, Yili Kazak Autonomous Prefecture	81°54′16.0590″	43°07′23.5493″	1226	8	YL

Note: Geographic coordinates and elevation were recorded during field surveys. The five exploratory groups were used for regional comparison and do not represent formally delimited biological populations.

Table S2. Information and screening outcomes for the 8 candidate InDel primer pairs

**Locus**	**Forward primer (5′–3′)**	**Tm (°C)**	**Reverse primer (5′–3′)**	**Tm (°C)**
1	TTTATGACAAAGCAATAATGGCT	57.0	CAGGGTATCTGAATGGAAGTAAA	56.0
2	TCTCCGCACCATTCCTTTAC	57.6	AGCATCTTAGCCGTCTTTGG	57.3
3	ATTACCCCATTGTGGATGCT	56.7	ACTCATGCGAAAAGTAACCTCTA	56.0
4	CAGTCATTTGCTGTGCTGAAG	56.7	AACCAGTGATAAGCCAACAATC	56.3
5	CATCAAGCAGGAGGAGAAGGA	59.3	TTCTCATGCGATGAACAAAGATA	58.0
6	CTCCGTGATGACCATAAAAAGATT	59.6	TTGAACCCCACAGAAAGGAGT	58.6
7	TCTCAAATAACGCGAAGGAAAT	58.9	CTACTCATCCCTTGCTTCAATTAA	58.1
8	CAAATACATACTTGTGAGGTCCG	57.0	GTAGAGGAAGCAAATTCAGGTGTA	57.8

Figure S1.Representative agarose gel electrophoresis profile for InDel genotype scoring

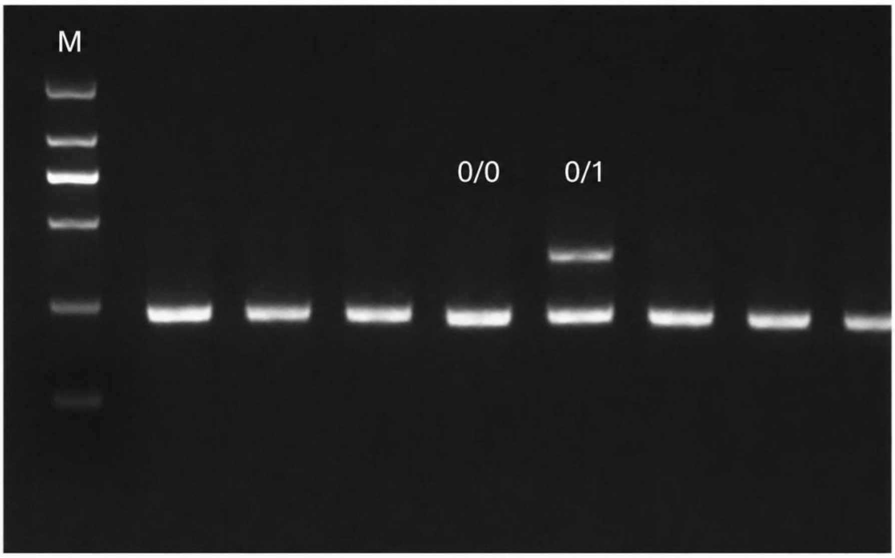

Note: M, DNA marker (100, 250, 500, 750, 1000, and 2000 bp); 0/0, short-fragment homozygote; 0/1, heterozygote with both short- and long-fragment alleles.

Figure S2. UPGMA tree of the 33 resequenced *C. acanthophylla* individuals based on the eight developed InDel markers. Colored ranges indicate the five exploratory analytical groups (AS, WS, JM, CJ, and YL). This analysis was used to compare InDel-based genetic relationships with the genome-wide SNP-based patterns obtained from the same 33 individuals.

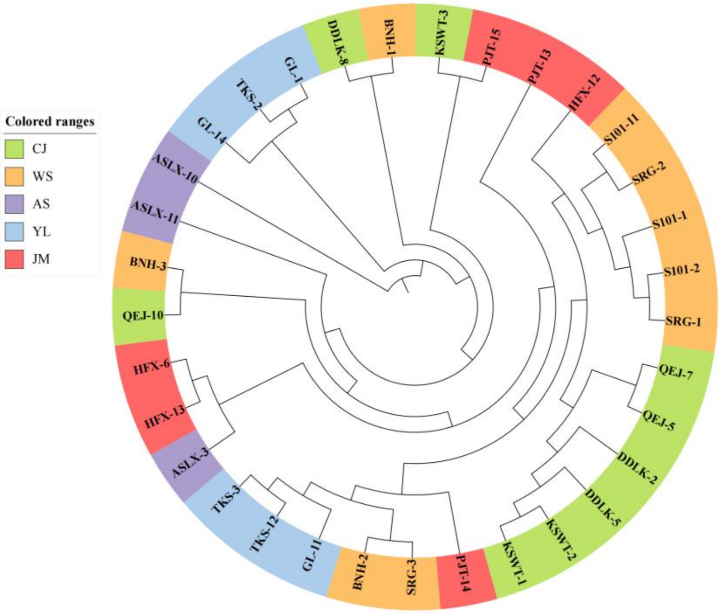

Figure S3. Evaluation of genetic clustering in STRUCTURE analysis. (a) Mean log probability of the data [LnP(K)] ± standard deviation across 15 independent runs for K = 1–10. (b) ΔK values calculated according to the Evanno method.

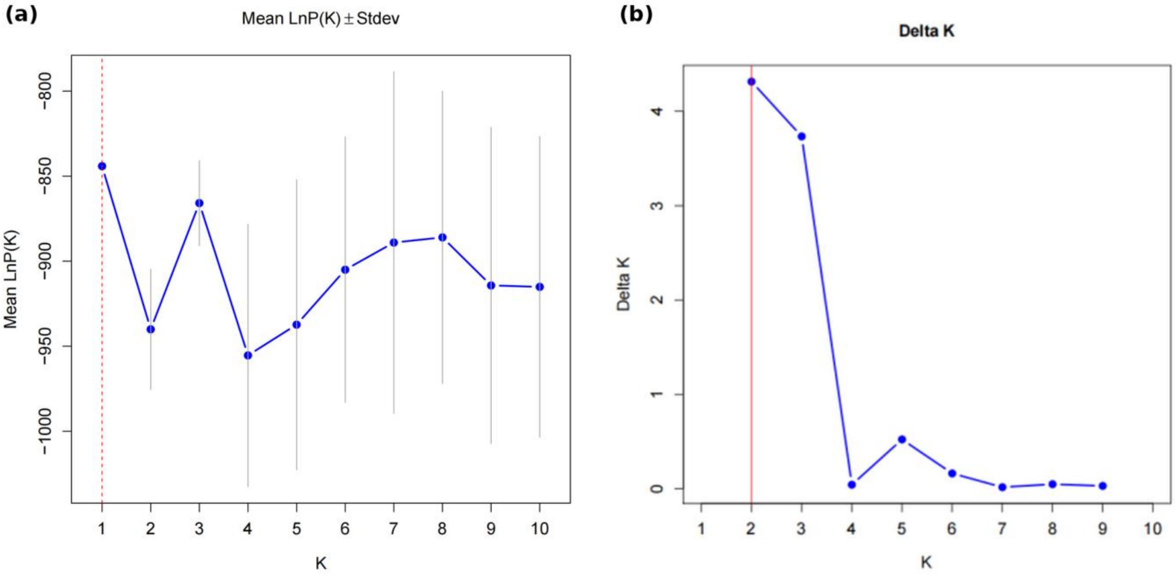
(a)
Mean LnP(K) ± Stdev
Mean LnP(K)
−800
−850
−900
−950
−1000
1
2
3
4
5
6
7
8
9
10
K
(b)
Delta K
Delta K
0
1
2
3
4
1
2
3
4
5
6
7
8
9
10
K
